# Tribological Properties of Laser Cladded Alloys for Repair of Rail Components

**DOI:** 10.3390/ma15217466

**Published:** 2022-10-25

**Authors:** Panahsadat Fasihi, Olivia Kendall, Ralph Abrahams, Peter Mutton, Cong Qiu, Thomas Schläfer, Wenyi Yan

**Affiliations:** 1 Department of Mechanical and Aerospace Engineering, Monash University, Clayton, VIC 3800, Australia; 2 Institute of Railway Technology, Monash University, Clayton, VIC 3800, Australia; 3 LaserBond Ltd., Cavan, SA 5094, Australia

**Keywords:** additive manufacturing, light rail, wear, rolling contact fatigue, microstructure

## Abstract

Tram or light rail systems are heavily relied upon for passenger transit; however, low-carbon steel grades commonly used in special trackwork, such as in switches, are prone to wear, rolling contact fatigue (RCF), and deformation under cyclic wheel–rail contact. To address this, laser cladding can be used to apply a metal coating to protect the underlying substrate and rebuild the worn rail profiles. Laser cladding may also be applied to remove cracking by rebuilding the rail head. The tribological characteristics of light rail components after laser cladding with Stellite 6 and a newly developed martensitic stainless steel were investigated, using roller-on-disc wear testing. Analysis of the microstructure, mechanical properties, and wear performance was undertaken to develop a comprehensive understanding of the influence of the laser cladding type on the wear and surface fatigue performance. Both cladding alloys significantly improved the tribological performance. These findings were compared to those for a laser cladded hypereutectoid rail type (reported in our previous study). It was found that laser cladding with a suitable alloy was an effective technique for improving the tribological properties, increasing the wear resistance, and increasing the retardation of cracking on both substrates. These findings suggest laser cladding may be used to repair light rail components, and this technique can be optimized to suit different rail grades. This makes laser cladding a flexible and versatile maintenance strategy, in both coating and repair applications, to prolong the operational lifetime of critical components for the railway industry.

## 1. Introduction

The railway industry faces the ongoing challenge of rapid rail deterioration due to high axle loading, increased rollingstock speeds, and greater service frequency to meet passenger demands. The operation of this critical infrastructure at these extreme conditions leads to wear, rolling contact fatigue (RCF), and plastic deformation, causing fracture and failures if expensive rail replacement procedures are not undertaken. These damages are particularly prevalent in light rail or tram components due to the lower carbon steel grades generally used for special trackwork and the presence of tight radius curves in urban environments. As light rail networks are generally associated with densely populated areas, the tracks often feature sharp curvatures and must withstand frequent acceleration and sudden braking, leading to premature wear and fatigue [1]. Standard maintenance procedures for light rail components use submerged arc welding techniques for repair applications [2]. Whilst this technology is widely implemented, welding repairs often result in large regions of softening and microstructural changes, which may be detrimental to the operational lifetime by increasing their susceptibility to fatigue and failure.

A new approach to rail maintenance uses laser cladding to deposit a metal at the rail surface in order to rebuild the worn profile or apply a coating with improved tribological properties. This is achieved by using a high energy laser that simultaneously melts a metallic powder and the rail substrate to metallurgically form a high quality coating layer. Laser cladding is a flexible process which can be modified to be undertaken in situ and requires lower heat inputs in comparison to standard welding-based techniques [3,4]. For these reasons, laser cladding has been widely investigated as a method to improve the wear and fatigue performance in railway applications. Lewis et al. [5] established that martensitic stainless steel and Stellite cladding alloys are effective in reducing deformation, fatigue, and wear. A study by Wang et al. [6] determined that laser cladding of heavy haul rail with a Co-Cr-based alloy effectively prevented adhesion and spalling wear mechanisms after rolling–sliding wear tests. Similarly, Seo et al. [7] undertook a study on cladding repairs for rails used in curved track and found that Stellite 21 achieved an 83% decrease in wear after twin-disc testing compared to the unclad rail. Hastelloy and Inconel 625 depositions also showed promising results, although the decrease in wear rate was less significant. Guo et al. [8] found that a single layer of cladding deposition on either rail or wheel decreased the extent of wear in both components by reducing the friction coefficient under rolling–sliding conditions. Zhu et al. [9] performed localized cladding repairs on rail and wheels using 316L, 410L, and SS420 steel grades. Each exhibited an improved wear resistance which was attributed to the increase in cladding hardness at the repair site. Investigations on laser cladding of hypereutectoid rails by Roy et al. [10] revealed SS420 to be the most effective cladding alloy for premium heavy haul rail in terms of volumetric wear rate. Whilst SS420 exhibited the best wear resistance, Stellite 6 was recommended as the optimum alloy for laser cladding repair, considering the combined microstructure, tribological, and mechanical performance results.

To date, a vast majority of laser cladding studies on rails have not considered the influence of the substrate rail grade. However Lu et al. [11] examined the wear behavior of a martensitic stainless-steel laser cladding deposition when applied to standard (R260) and lower hardness (R200) rail grades, using twin-disc tests. The authors found that, although the wear rate of the laser cladded R200 rail grade was significantly reduced compared to the unclad substrate, it was still greater than that of the R260 rail grade that was laser clad with the same cladding type. They attributed this to the decreased hardness of the cladding depositions.

Many countries, including Australia, France, Switzerland, and the United States, operate extensive light rail systems for passenger transport; therefore, expanding laser cladding technology to be compatible with light rail infrastructure is an essential aspect when developing a new maintenance strategy for the railway industry. Most of the published works in the literature discuss the laser cladding of railway components; however, as current maintenance techniques for light rail networks include submerged arc welding techniques, there is a need to develop and investigate a more robust maintenance technique, such as laser cladding for tram networks. In the current study, two cladding alloys, namely Stellite 6 and a newly developed martensitic stainless steel, were selected for laser cladding on a low-carbon steel grade used for fabrication of light rail switch blades. This study assesses the feasibility of using laser cladding technique for light rail applications, and also establishes the most suitable cladding alloys and laser cladding process parameters. Roller-on-disc wear testing was carried out to determine the optimum cladding alloys for these components. These findings were compared to those from previous tests on hypereutectoid rail grades used under heavy haul conditions to establish the influence of cladding alloys on wear performance. To investigate and elaborate on the tribological properties of different cladding depositions, microstructural analysis of the cladding layers and substrates, surface hardness measurements, and worn surface characterization using optical and scanning electron microscopic (SEM) imaging have been carried out. This will extend the use of laser cladding to a wider range of components and provide an alternative, efficient maintenance strategy to essential light rail networks.

## 2. Materials and Methods

### 2.1. Materials and the Laser Cladding Process

A low-carbon steel type (used for the fabrication of light rail switch blades), which is commonly used in passenger transport, was considered in this study. For comparison purposes, previous results [10,12] for a laser clad high-strength hypereutectoid premium rail grade (HE400) commonly used in heavy haul transportation was also considered.

To assess the influence of laser cladding materials on the wear performance of the light rail component, Stellite 6 and a new martensitic stainless-steel alloy (SS412) were selected for longitudinal single layer deposition on the light rail switch blade. The martensitic stainless-steel cladding material was developed through combining the standard Fe-based 410L and SS420 to ensure that this cladding alloy meets the microstructural and mechanical properties requirements of the low-carbon substrate material. This process was detailed in the studies by Fasihi et al. [12], and Kendall et al. [13], where the authors developed a similar Fe-based cladding alloy, namely SS415, from the two standard Fe-based alloys, 410L and SS420, for hypereutectoid rail applications. Several authors established that Co-Cr alloys, namely Stellite 6, are promising materials for cladding applications and are capable of improving the wear performance of premium train rail components [5,10,14,15].

The chemical compositions of the two substrates and the cladding alloys are summarized in Table 1. The average particle diameter of the deposited powders was 150 µm.

**Figure 1:**
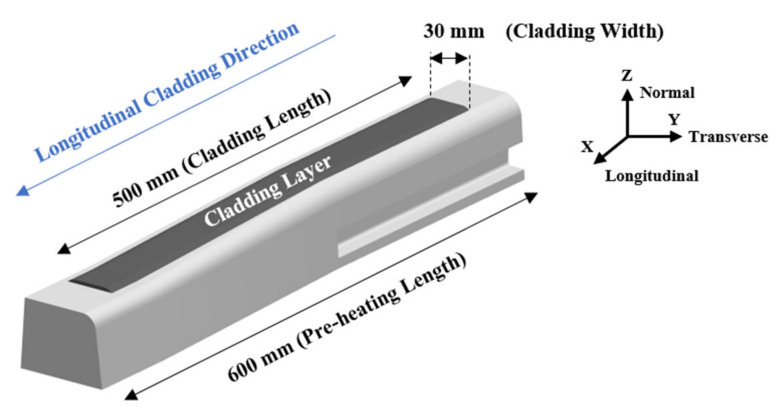
Schematic diagram of the laser cladded light rail.

For the light rail application, the laser cladding process was carried out by LaserBond Ltd. (Cavan (SA), Australia). The laser cladding was performed on a 600 mm long light rail section, as shown in Figure 1. The rail was preheated before a 2 mm thick single layer longitudinal deposition was applied over the middle 500 mm of the 600 mm rail section. As the light rail profile changed along the length of the blade, to ensure consistency, a cladding width of 30 mm is maintained along the rail head. Figure 1 shows the schematic diagram of the laser cladded light rail.

### 2.2. Roller-on-Disc Experiment and Analysis

#### 2.2.1. Sample Preparation

The light rail samples were cut into rectangular plates of 20 × 30 × 9 mm^3^, using electric discharge machining (EDM). The plates were then ground by 0.2 mm from the top surface to remove the rough cladded beads in order to provide a surface finish of 0.4 µm.

#### 2.2.2. Wear Test Apparatus

**Figure 2:**
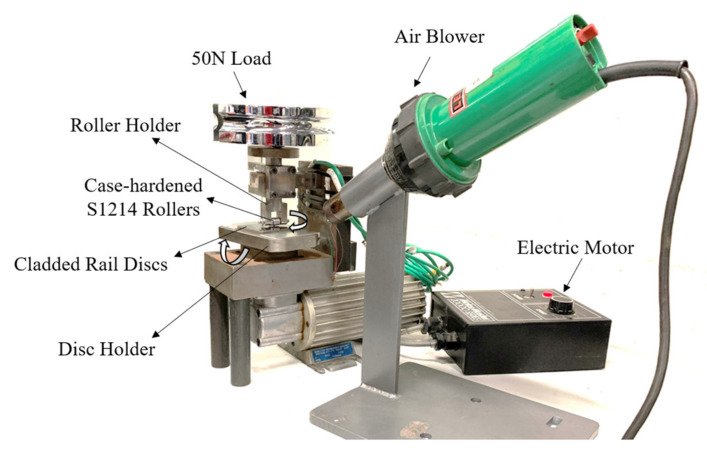
Roller-on-disc test apparatus and its components. The same facility was used in our previous study on the tribological properties of 410L, SS420, Stellite 6, and newly developed SS415 claddings [10,12].

A modified custom-made roller-on-disc machine, as shown in Figure 2, was used to determine the performance of the cladding alloys on both hypereutectoid and low-carbon steel substrates. During the test, the cladded rail specimen is in contact with the case-hardened S1214 rollers that have an average hardness of 950 HV5. It should be noted that the purpose of these tests was not to replicate the exact wheel–rail contact conditions; the test has been intentionally configured to examine the relative behavior of different cladding materials under more severe conditions. Table 2 summarizes the roller-on-disc test parameters. Wear tests were conducted under dry contact conditions, at ambient room temperature, with an applied load of 50 N. A detailed schematic diagram of the roller-on-disc tribometer and the detailed description of the test setup and parameters are presented in References [10,12].

#### 2.2.3. Evaluation of Hardness

The hardness of the non-clad and cladded plates was measured by a Vickers microhardness tester. The hardness indentations, both before and after testing, were performed using a 5 kg-f load, with a dwell time of 15 s, at six locations on the surface of the rail sections along the wear track.

The increase in hardness, based on the average of pre-test and post-test hardness values, was used to indicate the extent of work-hardening that occurred during the wear tests.

#### 2.2.4. Microstructural Analysis

The metallographic samples were obtained by sectioning the rails in the transverse direction. The sections were mounted, ground, polished, and etched with a 2% Nital etchant to expose the microstructure of the rail substrate. Kalling’s no. 2 solution (5 g CuCl_2_, 100 mL HCl, and 100 mL ethanol) was used to observe the cladding layer’s microstructure.

#### 2.2.5. Wear Measurements and Worn Surface Characterization

After the completion of the rolling cycles, the wear plates were removed from the roller-on-disc setup and cleaned in an ultrasonic cleaner with ethanol as solution. A compressed air gun was used to dry the surfaces. To image and characterize the wear scar, 3D laser optical profilometers, Olympus OLS4100 and OLS5100 (Olympus Corporation, Tokyo, Japan), were used. The analysis software, LEXT (version 2.1.2.8087), reconstructed a 2D profile of the laser-scanned worn area which was then used to calculate the wear volume loss. The wear volume loss was obtained by integrating the area of the wear scar profile and multiplying it by the circumferential length of the wear track.

The worn surfaces were then examined by using an Olympus GX optical microscope, with more detailed examination using either a Zeiss Merlin Gemini 2 Field Emission Scanning Electron Microscope (FESEM) (Carl Zeiss Microscopy GmbH, Jena, Germany), or a JEOL 7001F FEG Scanning Electron Microscope (JEOL Ltd., Tokyo, Japan).

## 3. Results and Discussion

### 3.1. Microstructure of the Non-Clad and Cladded Rails

**Figure 3:**
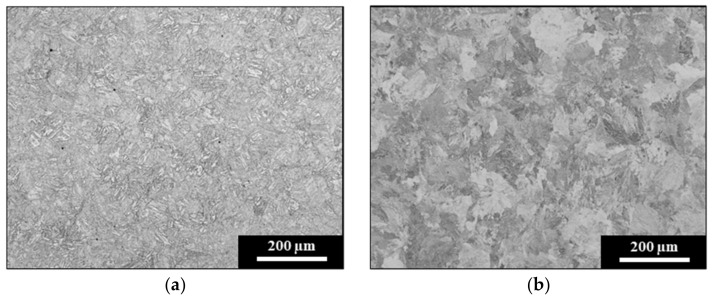
(**a**) Tempered martensitic microstructure of light rail, and (**b**) pearlitic microstructure of hypereutectoid rail.

The chemical composition of the light rail used as the laser cladding substrate is presented in Table 1, and indicates that this is a low alloy steel grade. Careful tempering procedures carried out by the rail industry increase the toughness and wear resistance of the rail by producing a tempered martensitic microstructure with an average hardness of 360 HV5, as shown in Figure 3a. In contrast, Figure 3b shows the microstructure of the hypereutectoid rail that was used for wear comparison in this paper. The high carbon content of this rail grade used by the heavy haul industry produces a fully pearlitic microstructure, resulting in a higher substrate hardness of 400 HV5. This hardness difference between the two rail substrate types is reflected in the wear plots obtained for the non-clad specimen, as shown in Section 3.2 below.

**Figure 4:**
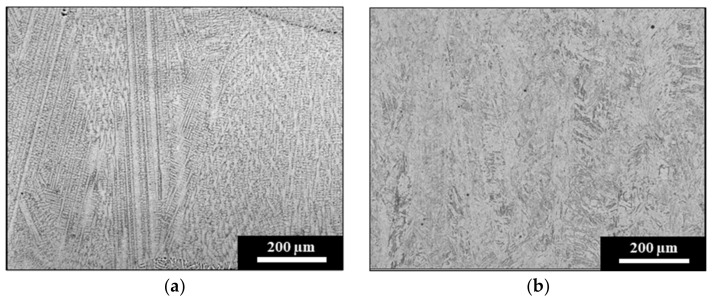
Laser cladding microstructure on light rail substrates from (**a**) Stellite 6 depositions containing cobalt-rich dendrites, and (**b**) SS412 depositions containing fully tempered martensite.

The microstructures of the Stellite 6 and stainless-steel SS412 depositions on the light rail substrate are shown in Figure 4. Laser cladding with Stellite 6 results in dendritic microstructure of cobalt-rich dendrites separated by a complex carbide phase, as shown in Figure 4a. The dendritic morphology and higher content of cobalt, carbon, and chromium compared to the light rail produce a deposition with a Vickers hardness of 450 HV5. This offers greater protection to the underlying and softer substrate. In comparison, SS412 produces a fully tempered martensitic cladding layer from the fusion boundary up until the cladding surface. The martensitic microstructure is shown in Figure 4b, indicating that this tempered morphology may be achieved with only a preheating procedure applied before the laser cladding process. The resultant hardness of the SS412 depositions on the light rail is 430 HV5. Although both the light rail and SS412 deposition have tempered martensitic microstructures, the significantly higher chromium content of the cladding layer enables a higher hardness to be produced compared to the steel substrate.

**Figure 5:**
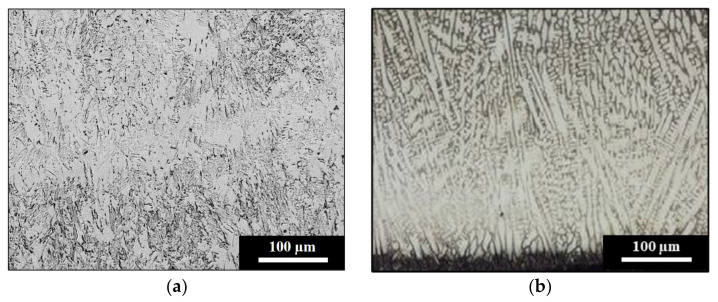
Cladding microstructure on hypereutectoid rail from (**a**) SS415 deposition containing partially tempered martensite, and (**b**) dendritic microstructure of the Stellite 6 cladding layer, published by Lai et al. [16].

The microstructures of the laser cladding deposits on the hypereutectoid rail are shown in Figure 5. SS415 is a martensitic stainless-steel, which, after the addition of a 350 °C post-cladding heat treatment, has resulted in a partially tempered martensitic morphology, as shown in Figure 5a. After this tempering process, the cladding deposition has a hardness of 530 HV5. Although the hypereutectoid substrate has a significantly higher carbon content than the cladding deposition, this increase in hardness is achieved through the combination of the partial dendritic morphology in the SS415 deposition and greater chromium content.

A similar dendritic microstructure to the Stellite 6 deposition on light rail was observed by Lai et al. [16] in Stellite 6 depositions on high-carbon rail. This is shown in Figure 5b and was reported to produce a cladding hardness of 441 HV5 [16]. The hardness values for the substrates and cladding depositions are presented in Table 3, which indicates the increased surface hardness achieved after laser cladding on both low- and high-carbon substrates and its influence on the cladding alloy and microstructure, not the substrate material.

### 3.2. Wear Performance of Non-Clad and Cladded Rails

**Figure 6:**
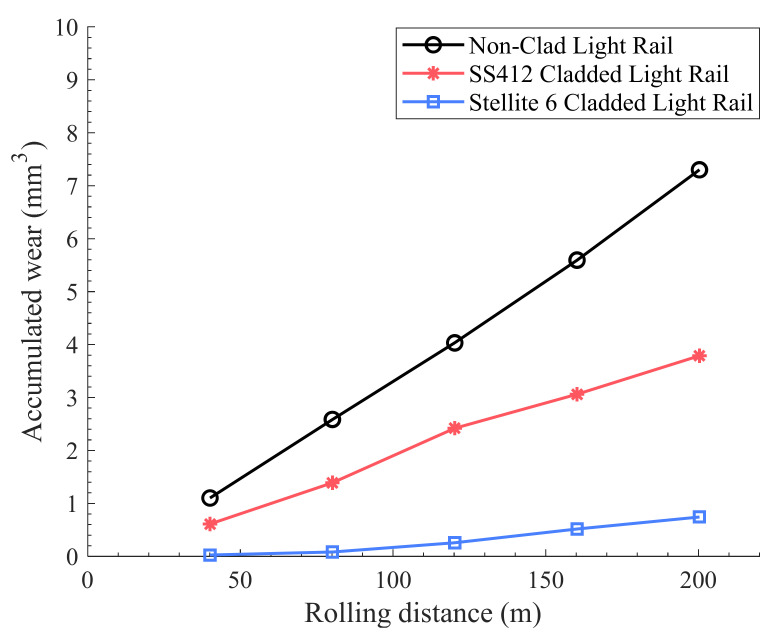
The accumulated wear performance of the non-clad and cladded light rails.

**Figure 7:**
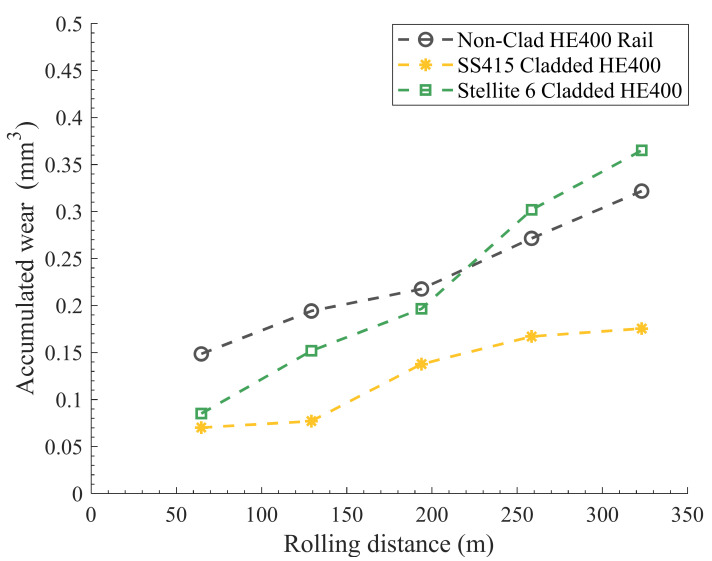
The accumulated wear performance of the non-clad and cladded hypereutectoid rails [10,12].

Figure 6 and Figure 7 show the comparison of the accumulated wear defined as the accumulated wear volume against the accumulated rolling distance for the non-clad and cladded light rails and hypereutectoid rails, respectively.

The non-clad, low-carbon light rail switch blade exhibits a significantly higher accumulated wear compared to the HE400 premium heavy haul rail. This lower wear resistance of the light rail can be attributed to its lower hardness and lower carbon content compared to the non-clad hypereutectoid rail; in addition, the difference in microstructure (i.e., tempered martensite vs. pearlite) may be a factor. In general, the wear performance of all cladding alloys decreased with increasing surface hardness; this trend was, however, more marked for the cladding on the light rail switch blade than for those on the hypereutectoid heavy rail.

For the light rail, the highest cladding layer hardness of 450 HV5 was observed with Stellite 6 cladding, which was followed closely by the hardness of 430 HV5 obtained with SS412 cladding. As shown in Figure 6 and Table 4, it is evident that cladding with Stellite 6 alloy is capable of reducing the volumetric wear by about 90%, compared to the non-clad light rail. This is due to the harder dendritic cladding microstructure with an interdendritic carbide phase providing a higher surface wear resistance. Moreover, as shown in Figure 6, the developed SS412 alloy resulted in a 48% decrease in the volumetric wear compared to the non-clad light rail. This cladding alloy exhibits a softer tempered martensitic microstructure similar to the substrate; therefore, it offers lower wear resistance under repeated loading cycles. Figure 6 demonstrates that single layer depositions of either Stellite 6 or stainless-steel alloy produce increased wear resistance on the light rail substrate.

Previous studies by Roy et al. [10], and Fasihi et al. [12] involved similar measurements on laser cladded hypereutectoid rail components. Those results are summarized in Figure 7. In comparison to the light rail switch blade, the higher carbon content of the hypereutectoid rail (approximately 5.5× of that of light rails) results in a harder pearlitic microstructure from the layers of ferrite and cementite, with an average hardness of 400 HV5, which reduced the wear compared to the non-clad light rail switch blade. For hypereutectoid rails, Stellite 6 resulted in the cladding layer hardness of approximately 441 HV5. SS415 achieved an average cladding layer hardness of 530 HV5. As shown in previously published results [10,12], the hardness variation within the cladding layer and HAZ were insignificant. As shown in Figure 7, the Stellite 6 cladding resulted in approximately a 15% increase in the volumetric wear in comparison to the non-clad hypereutectoid rail; however, this limited increase in the wear rate may be beneficial in reducing the crack growth rate [17,18]. Fletcher and Kapoor [19] showed that a limited extent of wear can reduce the crack propagation rate by altering the rail profile and moving the contact patch away from the highly stressed and crack-induced region. This phenomenon can be noticed in the optical images obtained for Stellite 6 cladding published by Roy et al. [10], where no observable cracking was detected on the Stellite 6 wear track.

A similar martensitic stainless-steel alloy (SS415) was developed particularly for hypereutectoid rail applications [12] and used for comparison to the performance of SS412 developed for the light rail switch blade in the current tests, due to the similarities in microstructure and composition. It can be observed that laser cladding using SS415 reduced the wear of hypereutectoid rail by about 78% compared to the non-clad rail. From Figure 7, it is evident that SS415 cladding exhibits a better wear performance, as compared to Stellite 6, for hypereutectoid rail applications.

The abovementioned results show that single layer laser cladding of Stellite 6 resulted in a reduction of accumulated wear on both substrates. However, the improvement in wear resistance over the non-clad substrate was greater for the light rail component in comparison to the same cladding material type on the high-carbon rail grade. This is due to the increased hardness of the Stellite 6 deposit in comparison to the light rail switch blade substrate. As the hypereutectoid rail is a harder steel grade, the increased wear resistance offered by Stellite 6 is less significant. The slight differences in the wear performance of the two Stellite 6 deposits can be attributed to the differences in the cladding parameters, as the two were prepared by different suppliers. It is worth noting that this variation was not the main focus of this study, and it is planned to be further investigated by the authors in future work. The current study mainly aimed to demonstrate the suitability and applicability of different laser cladded deposits on two different rail grades.

In comparison with the Stellite 6, the martensitic stainless-steel laser cladding on the light rail switch blade exhibited a lower decrease of 48%, in volumetric wear over the non-clad substrate, which is consistent with the difference in the pre-test hardness levels. To obtain a tempered martensitic microstructure in the SS415 cladding layer for the hypereutectoid rail, a post-cladding heat treatment process was required. This resulted in partial tempering of the cladding layer, resulting in a hardness of 530 HV5, which is still the highest deposit hardness used in this comparison. Although the wear rate was still lower than the non-clad substrate, fully tempering the deposit is expected to decrease the hardness and, hence, wear resistance, as demonstrated by the results obtained for the light rail material laser cladded with martensitic stainless-steel.

The difference between the pre-test and post-test hardness values was used to provide an indication of the extent of work-hardening. Several authors, namely Ball [20] and De Gee [21], showed that increased work-hardening can reduce the sensitivity of the material to surface cracking. Table 5 and Table 6 summarize these hardness values and their respective percentage increase for the light rail and high-carbon substrates, respectively. From these tables, it is evident that laser cladding, regardless of the cladding alloy and substrate used, resulted in a greater extent of work-hardening compared to the non-clad substrate, and this can be partly attributed to the higher wear rate of the latter in the tests.

For the light rail samples, both cladding materials resulted in similar levels of work-hardening. However, if the wear performance shown in Figure 6 above is taken into consideration, the overall performance of Stellite 6 cladding is more favorable for the light rail application. Similar results were obtained for the hypereutectoid rail cladded with Stellite 6, where a 34% increase in the hardness was achieved. For the harder hypereutectoid rail, a greater difference was observed between the Fe-based and Co-Cr-based claddings. From these results, it can be noted that cladding with Stellite 6 reduces the extent of surface cracking in comparison to the developed Fe-based alloys. These results are consistent with the optical and SEM images presented in Section 3.3.

### 3.3. Worn Surface Characterization of the Non-Clad and Cladded Rails

#### 3.3.1. Optical Micrographs of the Non-Clad and Cladded Rails

**Figure 8:**
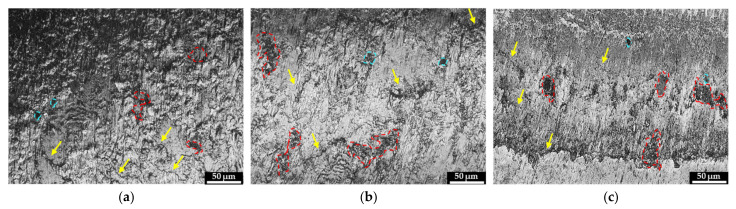
Optical micrographs of (**a**) non-clad light rail, (**b**) SS412, and (**c**) Stellite 6 cladding depositions on light rail specimen. Red boundaries represent regions of spalling, blue boundaries represent pitting, and yellow arrows indicate surface cracking.

**Figure 9:**
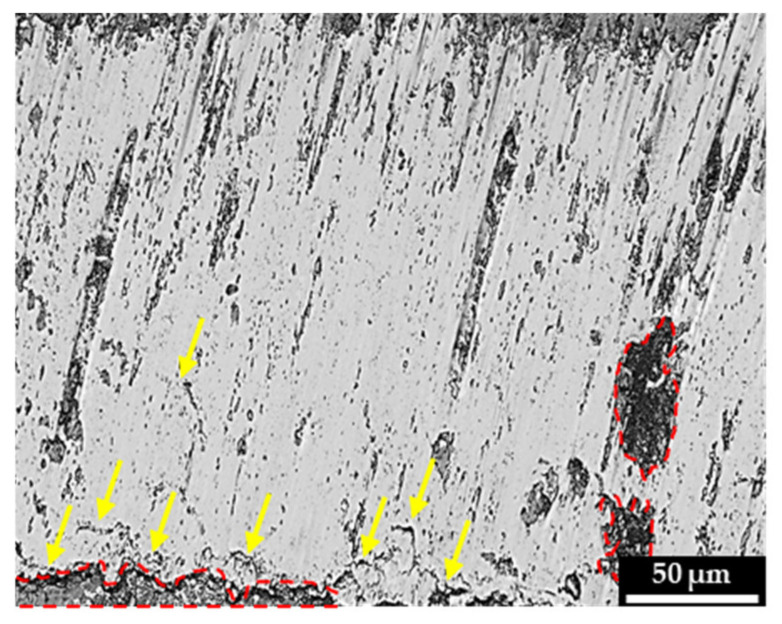
Optical micrograph of SS415 cladding depositions on hypereutectoid rail specimen. Red boundaries represent regions of spalling, and yellow arrows indicate surface cracking. The non-clad and Stellite 6 micrographs were published in a previous study by Roy et al. in Reference [10].

The optical micrographs of the worn non-clad and cladded light rail and hypereutectoid rail specimen are presented in Figure 8 and Figure 9, respectively. A range of different wear modes, namely adhesive and abrasive wear modes, are apparent in these micrographs. In these figures, the red boundaries represent regions of spalling, blue boundaries represent pitting, and yellow arrows indicate surface cracking.

From both figures, it is evident that laser cladding decreased the extent of surface damage and plastic deformation on both substrates. Despite this, as mentioned above, the performance of the cladded rails is highly dependent on the process parameters and the characteristics of the deposited material for the intended application. The non-clad light rail specimen shows a very rough plastically deformed surface with deep fatigue cracks that run along the surface; significant regions of spalling are also present on the tempered martensitic rail steel surface. It is worth noting that the SS412 deposition, shown in Figure 8b, still reduced the extent of rough worn surfaces caused by third-body abrasive wear particles, compared to the non-clad rail, shown in Figure 8a. As mentioned above, the SS412 deposition achieved a higher hardness compared to the light rail substrate, whilst retaining the tempered martensitic microstructure, which is desirable to avoid brittle fracture modes. Shallow cracks are visible at the SS412 deposition surface, indicating that fatigue cracking still occurs despite a higher wear resistance. With the Stellite 6 cladding, as shown in Figure 8c, a significant decrease in the extent of third-body abrasive wear modes, and shallower shorter surface cracking can be seen. This is attributed to the hard dendritic microstructure and carbide phase present in the cladding layer, which offers a greater resistance to crack propagation compared to tempered martensite, as was observed earlier in Figure 4b, when the light rail was cladded using SS412.

Similar to the light rail micrographs, a significant improvement in the worn surface morphology of cladded hypereutectoid rails can be observed. The worn surface morphologies of non-clad and Stellite 6 cladding were previously investigated by Roy et al. [10]. The authors showed that the non-clad specimen results in a large abrasive wear region and deep surface cracking [10]. As shown in Figure 9, an improved surface with the deposition of SS415 is achieved. SS415 deposition shows a much lower degree of surface cracking, which is also shallower in nature, and a smaller spalling region. Due to the post-cladding heat treatment undertaken on SS415, the microstructure contains partially tempered martensite, and the parent dendritic morphology is still visible. The higher carbon content offers an increased hardness, which improves the wear performance under rolling-contact loading. Martensitic stainless steel has also been reported to produce compressive residual stresses within the laser cladding deposition, thus increasing the energy required for crack propagation and retarding crack initiation [22]. In comparison, the SS412 deposition contains a fully tempered cladding microstructure with lower carbon content, hence showing a higher rate of wear than the other alloys. The Stellite 6 deposition on the high-carbon rail shows a significant improvement in the wear surface compared to the non-clad rail [10]. This is again attributed to the hard dendritic morphology of this cladding deposition, offering higher wear resistance, despite small regions of abrasive wear being visible on the laser clad surface after prolonged loading.

As stated above, the application of laser cladding significantly decreased the degree of surface damage on both light rail and hypereutectoid rail substrates. For both substrates, a lower degree of cracking was observed with Stellite 6 deposition, especially when deposited on low-carbon rail steel. The use of Fe-based SS412 and SS415 depositions on light rail and hypereutectoid rails, respectively, improved the extent of plastic deformation, but third-body abrasive wear modes, especially for light rails, were still present and dominant.

#### 3.3.2. SEM Images of the Non-Clad and Cladded Rails

**Figure 10:**
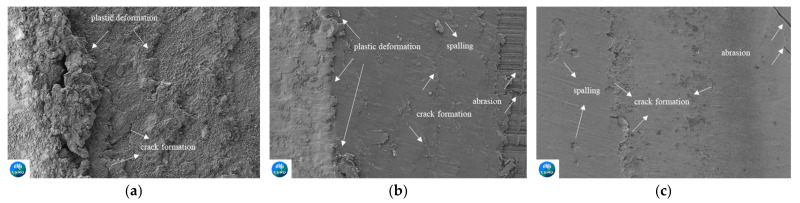
SEM images of (**a**) non-clad light rail, (**b**) SS412, and (**c**) Stellite 6 cladding depositions after roller-on-disc tests.

The SEM images of non-clad and cladded light rail samples are presented in Figure 10. The SEM images are consistent with the optical images and the abovementioned wear analysis. Figure 10a shows the plastically deformed non-clad worn light rail, for which the application of laser cladding resulted in an improved surface, lesser plastic deformation, and shallower cracks. The SS412 deposition, which is shown in Figure 10b, decreased the extent of plastic deformation and resulted in shallower and shorter regions of crack formation. As presented in Figure 10c, a more significant improvement was achieved with Stellite 6 deposition, for which only small regions of spalling, and much shorter, shallower cracking regions are observed. These results agree well with the hardness and wear analysis stated above.

**Figure 11:**
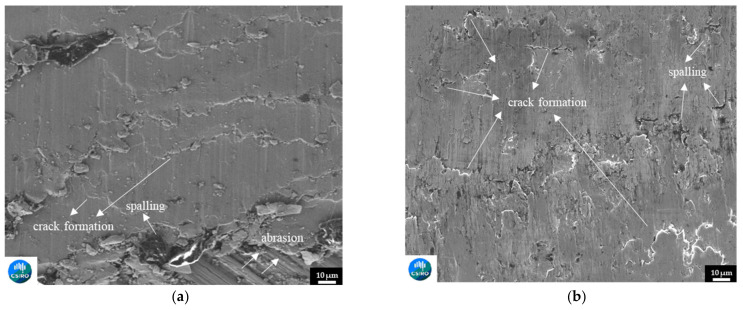
The worn surface morphology of the newly developed Fe-based alloys for (**a**) SS412 deposition on light rail and (**b**) SS415 deposition on hypereutectoid rail.

The worn surface morphologies of the newly developed Fe-based SS412 and SS415 alloys on light rail and hypereutectoid rail substrates are presented in Figure 11 (a and b), respectively. From the aforementioned wear performance, SS412 deposition resulted in about a 48% decrease in the volumetric wear of light rails, whilst SS415 deposition achieved the best performance for hypereutectoid cladding applications and achieved a 78% decrease in volumetric wear of hypereutectoid rails. This performance is observed in Figure 11b, where there are no regions of plastic deformation, and much shallower cracking is obtained with SS415 deposition, compared to the rougher worn surface obtained with SS412 deposition shown in Figure 11a.

## 4. Conclusions

The application of laser cladding for the maintenance and repair of worn light rail components has been experimentally investigated and compared against previously published results for laser cladded hypereutectoid rails. From this paper, it can be concluded that laser cladding is a feasible technique for the maintenance of different rail grades and complex components; however, the performance of the cladded components is highly dependent on the process parameters and characteristics of the deposited material for the intended substrate and application.

This study showed the effectiveness of the laser cladding process with desirable alloys in improving the tribological properties and reducing the extent of surface damage, plastic deformation, and cracking compared to the non-clad rails. For light rail applications, it was found that laser cladding with a Co-Cr-based alloy, compared to the newly developed Fe-based alloy, resulted in a more favorable tribological and material properties. The Stellite 6 deposition on light rail substrate resulted in about a 90% reduction in the volumetric wear, 26% increased hardness, and an improved worn surface morphology with much smaller and less significant regions of cracking. In comparison, a martensitic stainless-steel deposit (SS415) with post-cladding heat treatment was more effective at improving the wear resistance of a high-carbon rail substrate by increasing the surface hardness under rolling contact. These findings and this comparison suggest that this technique can be optimized to suit different rail grades, making laser cladding a flexible and versatile maintenance strategy in both coating and repair applications.

## Figures and Tables

**Table 1:** Chemical composition (wt.%) of the rail substrates and cladding alloys.

	Fe	Co	Cr	C	Mn	Si	Ni	Cu	Mo	V	Nb	Al	S	P	Ti
HE400 Rail	Bal	-	0.20	0.93	0.95	0.28	<0.01	-	<0.01	<0.01	<0.01	<0.01	0.014	-	-
Light Rail	Bal	-	0.46	0.17	1.20	0.24	1.01	0.10	0.48	0.03	0.02	0.06	<0.01	0.01	<0.01
SS415	Bal	-	12.4	0.13	0.85	0.48	0.21	0.04	0.03	0.01	<0.01	<0.01	0.01	0.02	<0.01
SS412	Bal	-	12.3	0.06	0.67	0.52	0.14	0.04	0.03	0.01	<0.01	<0.01	0.01	0.02	<0.01
Stellite 6	0.09	Bal	28.3	0.99	0.02	1.58	0.72	0.01	0.01	<0.01	<0.01	<0.01	0.01	0.03	0.01

**Table 2:** Roller-on-disc wear test parameters.

Load (N)	Rotational Disc Speed (RPM)	Wear Track Radius (mm)	Contact Conditions
50	28	5.45	Dry

**Table 3:** Average hardness (HV5) of cladding alloys and substrates.

Specimen	Light Rail (Substrate)	Hypereutectoid Rail (Substrate)
Non-Clad	360	400
Martensitic Stainless Steel	430	530
Stellite 6	450	441

**Table 4:** Total wear volume loss for non-clad and cladded light rails after the rolling distance of 200 m.

Specimen	Total Wear Volume Loss (mm^3^)	% Difference with Substrate
Non-Clad	0.2127 ± 0.007	N/A
SS412	0.1104 ± 0.016	−48.1
Stellite 6	0.0217 ± 0.006	−89.8

**Table 5:** Hardness for non-clad and cladded light rails before and after testing.

Specimen	Average Hardness before Testing (HV5)	Average Hardness after Testing (HV5)	Increase in Hardness (%)
Non-Clad	360.0 ± 16.8	419.1 ± 55.6	14.11
SS412	387.3 ± 57.7	516.7 ± 49.5	25.04
Stellite 6	416.9 ± 62.8	565.1 ± 63.9	26.22

**Table 6:** Hardness for non-clad and cladded hypereutectoid rails before and after testing [10,12].

Specimen	Average Hardness Before Testing (HV5)	Average Hardness after Testing (HV5)	Increase in Hardness (%)
Non-Clad *	388.4 ± 16.7	432.3 ± 16.7	10.15
SS415 **	520.2 ± 7.3	654.6 ± 15.4	20.56
Stellite 6 *	450.2 ± 16.7	683.3 ± 16.7	34.11

* The non-clad and Stellite 6 results were obtained from our previously published study in Reference [10]. ** The SS415 results were obtained from our previously published study in Reference [12].

## Data Availability

The raw and processed datasets generated and supporting the findings of this article cannot be shared at this time as the data also forms part of an ongoing study.
